# Handling incomplete correlated continuous and binary outcomes in meta‐analysis of individual participant data

**DOI:** 10.1002/sim.6969

**Published:** 2016-04-18

**Authors:** Manuel Gomes, Laura Hatfield, Sharon‐Lise Normand

**Affiliations:** ^1^ Faculty of Public Health and PolicyLondon School of Hygiene and Tropical MedicineLondonU.K.; ^2^ Department of Health Care PolicyHarvard Medical SchoolBoston02115MAU.S.A.; ^3^ Department of BiostatisticsHarvard T.H. Chan School of Public HealthBoston02115MAU.S.A.

**Keywords:** joint modelling, Bayesian analysis, fully conditional specification, multiple imputation, missing data, IPD meta‐analysis

## Abstract

Meta‐analysis of individual participant data (IPD) is increasingly utilised to improve the estimation of treatment effects, particularly among different participant subgroups. An important concern in IPD meta‐analysis relates to partially or completely missing outcomes for some studies, a problem exacerbated when interest is on multiple discrete and continuous outcomes. When leveraging information from incomplete correlated outcomes across studies, the fully observed outcomes may provide important information about the incompleteness of the other outcomes. In this paper, we compare two models for handling incomplete continuous and binary outcomes in IPD meta‐analysis: a joint hierarchical model and a sequence of full conditional mixed models. We illustrate how these approaches incorporate the correlation across the multiple outcomes and the between‐study heterogeneity when addressing the missing data. Simulations characterise the performance of the methods across a range of scenarios which differ according to the proportion and type of missingness, strength of correlation between outcomes and the number of studies. The joint model provided confidence interval coverage consistently closer to nominal levels and lower mean squared error compared with the fully conditional approach across the scenarios considered. Methods are illustrated in a meta‐analysis of randomised controlled trials comparing the effectiveness of implantable cardioverter‐defibrillator devices alone to implantable cardioverter‐defibrillator combined with cardiac resynchronisation therapy for treating patients with chronic heart failure. © 2016 The Authors. *Statistics in Medicine* Published by John Wiley & Sons Ltd.

## 1 Introduction

Patient‐centered outcomes research seeks, among other things, to understand which patients are most likely to benefit from available treatments, yet randomised controlled trials of new therapies are designed to answer primary efficacy questions, and often lack power to detect differential treatment effects across patient subgroups. Meta‐analysis of individual participant data (IPD) can aid the estimation of subgroup treatment effects by pooling information from multiple studies and using patient‐level data to improve precision. An important concern when pooling data across studies relates to partially or completely missing data for some studies. The main problem with missing data is that individuals with incomplete information tend to be systematically different from those with complete data, and inferences based on the complete cases may be misleading. Recent studies considered methods for handling predictors or confounders that are missing for some studies 1, 2. In this paper, we consider the dual problem of outcomes that are partially or completely missing for some studies, and multiple outcomes measured per study participant.

The approach taken to handling missing data should carefully consider the reasons that give rise to missing outcomes 3. Most published IPD meta‐analyses simply discard the observations with missing data in each individual study and report complete case analysis (CCA) 4. This ‘unprincipled’ approach assumes that the data are missing completely at random (MCAR); that is, missing values are independent of any observed or unobserved factors or may depend on variables included in the analysis model but are independent of other observed values (for example, other covariates or outcomes), a mechanism also known as covariate‐dependent missing completely at random (CD‐MCAR) 3. If patients with missing outcomes differ from those with complete information according to observed or unobserved characteristics (not adjusted in the analysis model), CCA will lead to inaccurate treatment effects. Principled approaches for handling missing data, such as multiple imputation (MI), maximum likelihood approaches, and full‐Bayesian analysis typically assume that data are missing at random (MAR). That is, the probability of missing data is independent of any unobserved values given the observed data. If the probability of missing data is associated with unobserved values, then the data are said to be missing not at random (MNAR).

The presence of multiple outcomes in randomised controlled trials is the norm rather than the exception 5. The assessment of alternative healthcare interventions is often based on multiple endpoints in order to characterise effects on a broad set of domains. For example, in medical devices studies, both patients and providers care about functional outcomes such as limits on daily activities, clinical endpoints such as time to progression or death, and device outcomes such as battery life and wear 6, 7. The multiplicity of outcomes has implications for the choice of statistical method for addressing the missing data. First, outcomes may be correlated within an individual participant; for example, patients with worse clinical endpoints may also have poorer functional outcomes. With incomplete correlated outcomes, the interest in addressing the missing data is greater compared with a single missing outcome as this allows us to appropriately handle the correlation between outcomes. Modelling correlated outcomes jointly can improve precision of treatment effects when compared with analysing them separately and enables joint inferences 8. In the context of IPD meta‐analysis, it is important to recognise that the relationships among multiple outcomes may arise at both the patient and the study level. Second, the probability that one outcome is observed (e.g. self‐reported quality‐of‐life) may depend, not only on patient and study‐level characteristics, but also on the other outcomes (e.g. clinical status). Third, population mean outcomes and associated variances, as well as prognostic relationships including treatment effects, typically differ across studies. Methods should appropriately address the between‐study heterogeneity when addressing the missing data. Fourth, differences in study design may cause some outcomes to be completely (or systematically) missing in some studies. In this case, leveraging information from the remaining studies to compensate for studies with systematically missing outcomes may be useful for inferences.

Our work is motivated by a meta‐analysis of randomised controlled trials that compare implantable cardioverter‐defibrillator (ICD) devices alone to ICD combined with cardiac resynchronisation therapy (CRT‐D). A few clinical factors are known to moderate the effectiveness of these devices, and recent investigations have focused on the question of whether men and women respond differently to these treatment options. Previous clinical trials have shown that these devices can improve survival, function and quality of life in patients with heart failure 9, 10. However, most of the US clinical trials that generated the evidence leading to device approval enrolled small numbers of women and each trial had its own restrictive entry criteria, which limited the ability to study broad sex heterogeneity in any individual trial.

In this paper, we consider two models for handling incomplete correlated mixed outcomes in IPD meta‐analysis: a joint hierarchical model 11 and a sequence of full conditional mixed models 12, 13. The way the joint model and the full conditional approach accommodate the correlation across outcomes and the between‐study heterogeneity when dealing with the missing data is distinct. The joint hierarchical model can incorporate the correlation structure among continuous and binary outcomes at both individual and study levels. Alternatively, specifying a sequence of univariate hierarchical regressions for each outcome conditional on all others avoids the complexity of specifying a joint hierarchical model, but the correlation between outcomes is not explicitly partitioned into patient and study‐level components.

Both the joint and full conditional models can be used with MI for handling the missing data 14. With MI, each missing value is replaced by a set of plausible values drawn from the posterior distribution of the missing data given the observed. MI allows the model for the missing data (imputation model) to be specified separately from the model for the complete data (substantive model). This paper considers MI to estimate the full conditional models using the chained equations approach, where the incomplete outcomes are imputed one at a time conditional on the observed data 15. For the joint model, we use a full‐Bayesian approach because current software options to implement MI to jointly impute continuous and binary hierarchical outcomes are limited. In the Bayesian framework, the missing values are simply nuisance parameters estimated along with the other unknowns (single step), ensuring internal consistency. Differences between MI and full‐Bayesian analysis for estimating the joint model (with missing outcomes) are trivial 16.

In non‐hierarchical settings, previous work has shown that imputing the missing outcomes iteratively is asymptotically equivalent to specifying a full joint distribution 13, 17, 30. That is, the imputed values from the chained equations converge to the values drawn from the posterior distribution implied by the joint model. However, it is unclear whether this equivalence holds in hierarchical settings such as meta‐analysis of patient‐level data. Hughes and colleagues 18 provide a condition – non‐informative margins – which together with compatibility 17, is sufficient for full conditional and joint models to produce values from the same predictive distribution. This condition requires that the marginal distribution of the conditioning variables provide no information about the parameters of the missing variable's imputation distribution. Specifying full conditional models of continuous and binary hierarchical variables that satisfy this condition is challenging 14, p 221]. With the joint model, all outcomes are included as responses, and it requires specifying an adequate covariance structure. Under this approach, the covariance of the incomplete variables is partitioned into individual and study‐level components, allowing for potentially complex correlation structures between outcomes 19. This paper investigates whether the joint hierarchical model yields more accurate treatment effect estimates compared with the full conditional approach in IPD meta‐analysis.

We assess the performance of the joint hierarchical model and a sequence of full conditional mixed models, across different circumstances in IPD meta‐analysis of correlated continuous and binary outcomes. We find that both approaches perform relatively well across all scenarios, although the joint model yields confidence interval (CI) coverage consistently closer to nominal levels and lower mean squared error, particularly when the outcomes are strongly correlated. Estimates of the average treatment effects of ICD versus CRT‐D for men and women are similar according to method, but the joint model provides more precise estimates. In the next section, we describe how each model addresses the correlation across the multiple outcomes and the between‐study heterogeneity when addressing the missing data. In Section 3, we describe our meta‐analysis of randomised controlled trials comparing implantable cardiac devices to treat heart failure, with missing binary and continuous outcomes (mortality, functional and quality‐of‐life endpoints). Section 4 outlines the simulation study. Sections 5 and 6 report the results of the simulations and case study. Finally, Section 7 discusses the findings and proposes some avenues for further research.

## 2 Methods

### 2.1 Joint hierarchical model

In this paper, we consider joint hierarchical models for a mixture of continuous and binary responses; extension to ordinal and categorical outcomes is relatively straightforward 11. When all incomplete outcomes are continuous, a multivariate normal model is the obvious choice. When incomplete outcomes are a mixture of continuous and binary, we can use a latent normal variable for each discrete outcome, and then model these jointly with the continuous outcomes using the multivariate normal model  20.

Let *Y*_1,*ij*_ and *Y*_2,*ij*_ be a continuous and binary outcome, respectively, for individual *i* in study *j*. Let *Z*_*ij*_ be a latent variable underlying *Y*_2,*ij*_, where *Y*_2,*ij*_ = 1 when *Z*_*ij*_ is positive, 0 otherwise. In addition, let *X*_*ij*_ be the set of predictors of the missing data. The unknown parameter of interest is the average effect of a treatment defined by *t*_*ij*_, where *t*_*ij*_ = 1 if the *i*th patient in study *j* is assigned to the new treatment, 0 otherwise. Then, we write our latent normal joint hierarchical model as

(1) $$\begin{array}{cc} Y_{1,\mathrm{ij}}=\mu_{1,\mathrm{ij}}+e_{1,\mathrm{ij}}\\ \;\;Z_{\mathrm{ij}}=\mu_{2,\mathrm{ij}}+e_{2,\mathrm{ij}}\;\;\;\;\;\;P(Y_{2,\mathrm{ij}}=1)=P\left(Z_{\mathrm{ij}}>0\right)\\ \left(\;\;\begin{array}{c} e_{1,\mathrm{ij}}\\ e_{2,\mathrm{ij}} \end{array}\;\;\right)∼N & \left[0,\Omega_{e}=\left(\begin{array}{cc} \sigma_{1}^{2} & \rho \sigma_{1}\sigma_{2}\\ \sigma_{2}^{2} \end{array}\right)\right]\;\text{and}\;\left(\begin{array}{c} u_{1,j}\\ u_{2,j} \end{array}\right)∼N\left[0,\Omega_{u}=\left(\begin{array}{cc} \tau_{1}^{2} & \phi \tau_{1}\tau_{2}\\ \tau_{2}^{2} \end{array}\right)\right] \end{array}$$

where *μ*_*k*,*ij*_=*β*_*k*,0_+*β*_*k*,1_ *t*_*ij*_+*β*_*k*,2_ *X*_*ij*_+*u*_*k*,*j*_ for *k* = 1,2. The covariance matrices ***Ω***_*e*_ and ***Ω***_*u*_ govern the individual and study‐level variability, respectively. For identification, the variance of the discrete outcome, $\sigma_{2}^{2}$, is fixed to 1. The correlation between responses at the individual and study‐level is explicitly recognised via the parameters *ρ* and *ϕ*, respectively. Model (1) assumes that only the random intercepts (*u*_1,*j*_, *u*_2,*j*_) vary across studies. Models that include heterogeneous treatment effects are considered later in this paper.

The joint model is estimated using a full‐Bayesian approach. With Bayesian analysis, we need a joint likelihood for the complete data, *P*(*Y*|*X*,*θ*), and a prior distribution for the unknown parameters, *P*(*θ*), including the treatment effect. Conditional on the observed outcomes, *Y* ^*obs*^, the posterior distribution is defined as *P*(*θ*|*X*,*Y* ^*obs*^). While full‐Bayesian analysis can allow additional information to enter via the prior distributions, we do not exploit this feature here, and instead considered vague or weakly informative priors throughout 21, 22. Because the focus of the paper is on incomplete outcomes, we do not need a separate missingness model for the missing values, *Y* ^*mis*^, provided that data are MAR. For this to hold, *X* in model (1) must include all variables that predict the missing values and the probability of missingness. Details about a Markov Chain Monte Carlo algorithm to estimate the parameters of interest are provided in Supporting Information, Appendix A.

### 2.2 Fully conditional specification

Under the fully conditional specification (FCS), the multivariate model is specified by a series of conditional models, one for each incomplete outcome. Using the same continuous and binary outcomes earlier (*Y*_1,*ij*_, *Y*_2,*ij*_), we define our two full conditional models as

(2) $$\begin{array}{cc} Y_{1,\mathrm{ij}}|Y_{2,\mathrm{ij}} & ∼N\left(\mu_{1,\mathrm{ij}}+\alpha_{1}Y_{2,\mathrm{ij}},\sigma_{1}^{2}\right)\\ Y_{2,\mathrm{ij}}|Y_{1,\mathrm{ij}} & ∼\text{Ber}\left\{\frac{\exp\left(\mu_{2,\mathrm{ij}}+\alpha_{2}Y_{1,\mathrm{ij}}\right)}{1+\exp\left(\mu_{2,\mathrm{ij}}+\alpha_{2}Y_{1,\mathrm{ij}}\right)}\right\} \end{array}$$

where *μ*_1,*ij*_ and *μ*_2,*ij*_ are defined previously. Model (2) allows for the between‐study heterogeneity by including study‐specific random intercepts (*u*_*k*,*j*_). The parameters governing the relationships between the two outcomes are *α*_1_ and *α*_2_.

Multiple imputation by chained equations 15 is an attractive approach for addressing missing data under the fully conditional approach. With MI, each missing value is replaced with a set of M plausible values. These values are drawn, in a Bayesian manner, from the conditional distribution of the missing observations given the observed data so that the set of imputed values reflects the uncertainty associated with both the missing data and the estimation of the parameters in the imputation model. Hence, MI is regarded as an approximate Bayesian method. A distinct feature of MI compared with the full‐Bayesian analysis is that the model for the missing values is specified separately from the substantive model for estimating the parameters of interest.

After constructing *M* multiple imputed data sets, we apply the following substantive model to each dataset:

(3) $$\begin{array}{c} Y_{1,\mathrm{ij}}=\beta_{1,0}+\beta_{1,1}t_{\mathrm{ij}}+\beta_{1,2}X_{\mathrm{ij}}+u_{1,j}+e_{1,\mathrm{ij}}\\ \text{probit}(Y_{2,\mathrm{ij}}=1)=\beta_{2,0}+\beta_{2,1}t_{\mathrm{ij}}+\beta_{2,2}X_{\mathrm{ij}}+u_{2,j}+e_{2,\mathrm{ij}}\\ e_{\mathrm{ij}}∼N(0,\Omega_{e})\;u_{j}∼N(0,\Omega_{u})\;. \end{array}$$

The parameters of interest such as the treatment effects *β*_1,1_ and *β*_2,1_ are combined using Rubin's rules 3, to properly reflect the variation both within and between imputations.

The chained equations algorithm to generate M imputed datasets is described in Supporting Information, Appendix B. Unlike the joint model, each incomplete outcome is imputed at a time, conditional on all other variables in the imputation model (including the other outcomes). It is unclear whether the stationary distribution generated by this fully conditional approach based on Model (2) asymptotically approximates the posterior predictive distribution implied by the joint hierarchical Model (1). Our simulation study assesses the relative performance of these alternative approaches to handle the missing data when estimating treatment effects in IPD meta‐analysis.

### 2.3 Complete case analysis

A relatively simple approach to missing data is to apply the substantive model (3) to the complete data, discarding patients for whom at least one outcome is missing. This approach makes the strong assumption that missingness is unrelated to unobserved values, conditional on the variables included in the substantive model. However, patients with incomplete data may differ from those with complete information according to other observed or unobserved characteristics, and inferences based on the complete cases may be biased. In addition, CCA can lead to large proportions of patients being dropped, or some studies being discarded altogether (in the case of systematically missing outcomes). For completeness, we consider CCA as a worst‐case alternative prone to both bias and inefficiency.

## 3 Motivating example

Our motivating data come from five randomised controlled trials of implantable cardiac devices used to treat heart failure obtained from the Center for Devices and Radiological Health, the United States Food and Drug Administration. ICD devices can sense abnormal heart rhythms and deliver a high‐voltage shock to correct an arrhythmia in order to prevent cardiac arrest and sudden death. These devices are often combined with additional pacing leads for patients who also require regular pacing. For some patients, this means bi‐ventricular pacing or cardiac resynchronisation therapy (CRT). We combined data from studies that compared an ICD alone to ICD with CRT function added (CRT‐D).

We want to compare treatment effects on multiple outcomes, not just a single endpoint. These comprise mortality, improvement in physician‐rated disease severity on the New York Heart Association (NYHA) scale (1 if NYHA class improved, 0 otherwise), a functional measure of the distance walked in 6 min, and quality‐of‐life measured by the Minnesota Living with Heart Failure questionnaire. In particular, we would like to investigate whether men and women respond differently to treatment. Combining data from across studies enlarges the sample size, allowing more precise subgroup treatment effect estimates. This is particularly true in these trials, which enrolled small numbers of women. Each individual study specified different times at which functional and quality‐of‐life outcomes were measured. Here, we considered 1‐year endpoints whenever available (three studies), and 6‐month otherwise (two studies).

New York Heart Association, 6‐min walk and quality of life outcomes are not defined for patients who die before the time designated for their outcomes to be measured. An approach commonly used in longitudinal settings is to jointly model the survival time with outcomes censored due to death. In our setting, outcomes are measured only at two time points, and these models are not feasible. Given the low proportion of mortality (4%) and to be consistent with the trials' primary analysis, we assumed that when patients die they have the same outcomes measured in the previous time point. For example, if a patient dies between 6 and 12 months, the 1‐year analysis considers the outcome values measured at 6 months. As a sensitivity analysis, we set all those outcomes to their ‘worst’ values (zeros in all cases) in the presence of mortality. In other words, we assumed that deceased patients' quality of life is zero, the NYHA binary outcome is zero (no improvement in NYHA class compared with baseline) and number of metres walked in 6 min is also zero. Not surprisingly, the results are very similar to those presented in Figure 3 given the small proportion of censored outcomes due to death.

Table 1 describes the main characteristics of these five studies, by intervention group. The proportion of missing data varied across studies. While mortality was fully observed for most studies, all other outcomes had high proportions of missing values per study ranging from 5% to 100%. Quality‐of‐life as measured by the Minnesota Living with Heart Failure questionnaire was completely missing for two studies; one study did not include this outcome in the protocol, and another used a different quality‐of‐life questionnaire. The proportion of complete cases, that is, patients who had all outcomes observed was 52%. The proportion of female patients in most trials was below 20% (sex indicator was fully observed).

**Table 1:** Descriptive statistics of the five randomised controlled trials comparing alternative implantable cardiac devices to treat chronic heart failure (*N* = 5273).

	1		2		3		4		5	
Study	ICD	CRT‐D	ICD	CRT‐D	ICD	CRT‐D	ICD	CRT‐D	ICD	CRT‐D
No. of patients (N)	245	245	283	272	904	894	191	419	731	1089
Proportion female (%)	17	15	18	20	19	15	20	21	24	25
Proportion observed (%)										
Mortality	87	89	100	100	100	100	100	100	97	99
NYHA class	47	45	92	90	83	84	98	97	89	83
6‐min walk	38	37	89	88	71	74	96	94	83	89
Quality‐of‐life	42	41	89	90	0	0	95	93	0	0
Correlation across outcomes					
Mean [min,max]	0.18	0.20	0.22	0.19	0.16	0.20	0.26	0.21	0.15	0.17
	[0.09, 0.24]	[0.05, 0.27]	[0.04, 0.35]	[0.08, 0.32]	[0.06, 0.22]	[0.08, 0.25]	[0.09, 0.39]	[0.07, 0.33]	[0.03, 0.22]	[0.04, 0.24]

ICD, implantable cardioverter‐defibrillator; CRT‐D, cardiac resynchronisation therapy; NYHA, New York Heart Association.

The range of correlations between outcomes was similar across studies and treatment arms but differed across outcomes. For example, mortality was weakly associated with any of the other outcomes (correlation coefficient ranged from 0.03 to 0.17), but the functional outcomes were more strongly correlated between them and with quality‐of‐life (0.18 to 0.39). The proportion of the total variation at the study level was relatively higher for the quality‐of‐life outcome (intra‐study correlation coefficient of 0.14) than the other outcomes (intra‐study correlation coefficient below 0.10).

## 4 Simulation study

### 4.1 Data‐generating process

The simulation study was designed to replicate typical features of IPD meta‐analyses (including those of our motivating example). Let the active treatment group be defined by *t*_*ij*_=1 and the control group by *t*_*ij*_=0. We simulate continuous and binary variables from a joint distribution using copulas, a useful method for constructing a joint distribution given the marginals 23. For the *i*th individual in study *j*, let $Y_{1,\mathrm{ij}}∼N\left(\mu_{1,\mathrm{ij}},\sigma_{1}^{2}\right)$ be the continuous outcome and *Y*_2,*ij*_∼Bernoulli(*π*_*ij*_) be the binary outcome. Drawing from a multivariate normal, converting to the uniform CDF scale, and then inverting the CDFs of both marginals, we obtain a draw from a joint distribution. Specifically, the parameters of the two marginals are

(4) $$\begin{array}{cc} \mu_{\mathrm{ij}} & =1+1t_{\mathrm{ij}}+0.5X_{1,\mathrm{ij}}+0.5X_{2,\mathrm{ij}}+u_{1,j}\\ \text{logit}(\pi_{\mathrm{ij}}) & =-0.5+0.1t_{\mathrm{ij}}+0.2X_{3,\mathrm{ij}}+0.2X_{4,\mathrm{ij}}+u_{2,j} \end{array}$$

$$\begin{array}{c} \left(\begin{array}{l} u_{1,j}\\ u_{2,j} \end{array}\right)∼N\left[\left(\begin{array}{l} 0\\ 0 \end{array}\right),\left(\begin{array}{ll} 1 & \phi \\ \phi & 1 \end{array}\right)\right] \end{array}$$

where *X*_*k*,*ij*_∼MVN(**0**,**Σ**) for *k* = 1,…,4 and **Σ** is the *k* × *k* identity matrix. Model (4) produces outcomes that are correlated both within each individual participant (*ρ*) via the copula, and within study (*ϕ*) via the random effects (*u*_1,*j*_ and *u*_2,*j*_). We assume a unit variance for the continuous variable, $\sigma_{1}^{2}=1$, and chose regression coefficients so that the correlation between covariates and outcomes was about 0.5. The true treatment effects are 1 for the continuous outcome and 0.1 for the binary outcome. Note that the linear predictor from a probit model (used to estimate the substantive model) is 0.6 that from the logit model and hence the true value under the probit model is 0.06 14, p. 94–95].

Next, we describe the framework to simulate the missing data. We considered scenarios where outcomes are missing for some patients within each study (sporadically missing), and missing for all patients in some studies (systematically missing). Let *P*(*R*_*k*,*ij*_=1) denotes the probability that the *k*th response for individual *i* in study *j* is missing. We simulated sporadically missing outcomes under MAR as

(5) $$\text{logit}P\;\left(R_{k,\mathrm{ij}}=1|X_{k,\mathrm{ij}},Y_{-k,\mathrm{ij}}\right)=\theta_{k,0}+\theta_{k,1}X_{k,\mathrm{ij}}+\theta_{k,2}Y_{-k,\mathrm{ij}}$$

that is, the probability of missingness may be related to observed covariates *X*_*k*,*ij*_ as well as the other response, *Y*_−*k*,*ij*_. We chose *θ*_*k*,0_ and *θ*_*k*,1_ to produce correlation of about 0.3 between the missingness indicator (*R*_*k*,*ij*_) and the predictors (*X*_*k*,*ij*_). For scenarios where the probability of observing one outcome depends on the other outcome (*θ*_*k*,2_≠0), we have randomly split the sample in two and applied model (5) to each sub‐sample (one for each outcome *k*) in order to preserve MAR. This ensures that, for each sub‐sample, the *P*(*R*_*k*,*ij*_=1|*X*_*k*,*ij*_,*Y*_−*k*,*ij*_) only depends on observed values of *Y*_−*k*,*ij*_. For systematically missing data, we set binary outcomes to missing for all individuals in some studies.

Table 2 summarises the parameter values across the scenarios. We contrast scenarios with few studies to those with more studies and thus more plausible asymptotics for the study‐level random effects. We consider alternative levels (low versus high) of correlations between outcomes at the individual (*ρ* = 0.2 and 0.7) and study‐level (*ϕ* = 0.1 and 0.3). We compare scenarios with proportions of missing data that are low (20%) or high (50%), and scenarios in which missingness depends only on observed covariates or also on the other endpoint. In addition, we considered systematically missing data for the binary outcome; for these scenarios, we set sporadically missing values to ‘low’ (20%) for the continuous outcome and then set the binary outcome to be completely missing in a few or many studies. The total sample size was 3000 across all scenarios, with a balanced treatment allocation between the two arms.

**Table 2:** Factors and their chosen levels varying across the different scenarios.

Parameter	Values
Sample size	(i) 20 studies, 150 individuals/study
	(ii) 5 studies, 600 individuals/study
Correlation between responses	Study level: (i) *ϕ* = 0.1; (ii) *ϕ* = 0.3
	Individual level: (i) *ρ* = 0.2; (ii) *ρ* = 0.7
Missingness predictors	(i) Covariates *X*_*k*,*ij*_
	(ii) Covariates *X*_*k*,*ij*_ and the other outcome *Y*_−*k*,*ij*_
% missing across studies	(i) 20*%* and (ii) 50*%*
Systematically missing data	set binary outcome to be completely missing
	for *J* studies such that overall % missingness
	is around (i) 20% and (ii) 50%

### 4.2 Implementation

For the FCS–MI approach, we conducted 10 imputations and 10 iterations between each imputation. The overall MI procedure was implemented in R using the *mice* package. To impute continuous outcomes, we used the *2l.norm.me* method 1 which allows for both sporadically and systematically missing data. Similarly, we used the *2l.bin* algorithm 24 for imputing binary outcomes. We considered two fully conditional models: one which does not include the other outcomes in the imputation model (Fully Conditional 1), that is, assume *α*_1_=*α*_2_=0(Model (2)); and one that includes the other outcomes (Fully Conditional 2). We hypothesise that even when the probability of one outcome being missing is not related to the other outcome (*θ*_*k*,2_=0, model (5)), including the other endpoint in the imputation model may improve CI coverage given the correlation between the outcomes. After imputation, we applied the analysis model (3) to each multiple imputed dataset using MLwiN via interface with the *R2MLwiN* package in R, which facilitates the estimation of joint multilevel models with mixture types of responses 25.

The joint hierarchical model, assuming vague priors, is described in Supporting Information, Appendix C. We considered 50 000 Markov Chain Monte Carlo iterations, after which convergence was good for both regression coefficients and variance/covariance parameters (Gelman–Rubin scale reduction factor was smaller than 1.1). The Bayesian approach was implemented in JAGS 26, which can be used via interface with R *rjags* package. The joint hierarchical model could also be used with MI, but joint imputation models for mixed hierarchical responses require the use of specialist software such as Realcom‐impute 27. This stand‐alone package is computationally slow, and the existing version cannot be automatised, making it impractical for our simulation study. For each scenario, we apply the methods to 1000 simulated datasets and compare the approaches according to bias, root mean squared error (rMSE) and joint CI coverage 28 for the treatment effects on continuous and binary outcomes.

## 5 Results

Table 3 shows the results for scenarios where the probability of missingness depends only on the covariates (sporadically missing outcomes only). Across these scenarios, we varied the individual‐level correlation (but fixed study‐level correlation, *ϕ* = 0.1), the proportion of missing data and the number of studies. Both the joint model and FCS approaches provided treatment effect estimates close to the truth and joint CI coverage near the nominal level (95%). Including the other outcome in the imputation model (Fully Conditional 2) did not improve performance when compared with Fully Conditional 1, even when the outcomes are highly correlated. The joint model provides slightly lower rMSE (closer to that of the full data analysis). CCA was biased and had poor (below 0.8) CI coverage, even with little missing data, many studies and weak correlation between outcomes.

**Table 3:** Percent bias, rMSE and joint CI coverage for the estimated treatment effect on continuous (*β*_1,1_) and binary (*β*_2,1_) outcomes when missingness depends on observed covariates only (sporadically missing outcomes).

			Bias (%)		rMSE		Joint CI
Correlation (rho)	*%* Missing	Method	*β*_1,1_	*β*_2,1_	*β*_1,1_	*β*_2,1_	Coverage
20 Studies							
Low (0.2)	20	Full data	0.0	0.9	0.040	0.076	0.952
		Complete‐cases	7.1	23.1	0.086	0.100	0.747
		Fully Conditional 1	0.1	2.0	0.043	0.083	0.954
		Fully Conditional 2	0.1	1.4	0.042	0.082	0.955
		Joint model	0.0	1.3	0.037	0.080	0.949
	50	Full data	0.1	1.1	0.040	0.076	0.952
		Complete‐cases	9.9	35.2	0.121	0.127	0.695
		Fully Conditional 1	0.2	3.9	0.051	0.102	0.959
		Fully Conditional 2	0.3	3.6	0.050	0.099	0.957
		Joint model	0.1	2.9	0.037	0.080	0.949
High (0.7)	20	Full data	0.1	1.0	0.040	0.075	0.958
		Complete‐cases	7.3	23.3	0.085	0.093	0.582
		Fully conditional 1	0.1	4.5	0.042	0.082	0.962
		Fully conditional 2	0.1	3.7	0.042	0.080	0.956
		Joint model	0.0	2.6	0.038	0.080	0.960
	50	Full data	0.0	1.2	0.040	0.075	0.958
		Complete‐cases	10.1	34.9	0.121	0.128	0.503
		Fully Conditional 1	0.1	7.3	0.052	0.101	0.945
		Fully Conditional 2	0.1	5.9	0.048	0.099	0.946
		Joint model	0.0	3.9	0.040	0.093	0.957
Five studies							
Low (0.2)	20	Full data	0.2	1.8	0.042	0.079	0.949
		Complete‐cases	6.5	30.3	0.082	0.097	0.751
		Fully Conditional 1	0.3	7.4	0.047	0.085	0.950
		Fully Conditional 2	0.3	7.2	0.046	0.085	0.948
		Joint model	0.1	2.3	0.040	0.083	0.954
	50	Full data	0.2	1.6	0.042	0.079	0.949
		Complete‐cases	9.8	30.4	0.123	0.131	0.731
		Fully Conditional 1	0.3	7.8	0.052	0.104	0.944
		Fully Conditional 2	0.2	6.5	0.051	0.100	0.941
		Joint model	0.1	2.4	0.043	0.094	0.946
High (0.7)	20	Full data	0.3	1.9	0.042	0.079	0.961
		Complete‐cases	6.4	31.1	0.081	0.097	0.600
		Fully Conditional 1	0.4	12.4	0.045	0.085	0.941
		Fully Conditional 2	0.3	7.6	0.043	0.085	0.947
		Joint model	0.2	3.2	0.040	0.084	0.950
	50	Full data	0.3	1.8	0.042	0.079	0.961
		Complete‐cases	9.7	41.2	0.121	0.133	0.532
		Fully Conditional 1	0.4	16.6	0.053	0.106	0.939
		Fully Conditional 2	0.3	8.3	0.050	0.101	0.942
		Joint model	0.2	2.4	0.042	0.096	0.951

Study‐level correlation is fixed across these scenarios: *ϕ* = 0.1. Fully Conditional 1: imputation includes covariates only; Fully Conditional 2: imputation uses covariates and the other outcome.rMSE, root mean squared error; CI, confidence interval.

**Figure 1:**
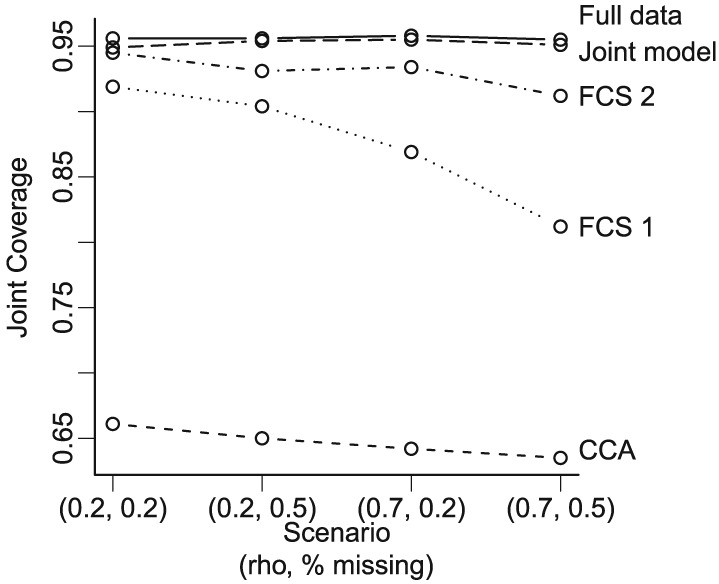
Joint confidence interval coverage of treatment effects on both outcomes when the probability of observing one outcome depends on both the observed covariates and the other outcome (sporadically missing data). Study‐level correlation is fixed across these scenarios: *ϕ* = 0.1. The lines are used to improve visualisation but do not reflect an increase of a single parameter in the *x*‐axis. FCS, fully conditional specification; CCA, complete case analysis.

**Figure 2:**
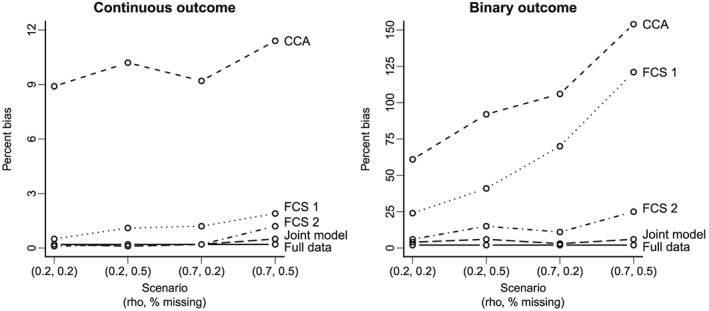
Percent bias of treatment effects on both outcomes when the probability of observing one outcome depends on both the observed covariates and the other outcome (sporadically missing outcomes). Study‐level correlation is fixed across these scenarios: *ϕ* = 0.1. The lines are used to improve visualisation but do not reflect an increase of a single parameter in the *x*‐axis. FCS, fully conditional specification; CCA, complete case analysis.

Figures 1 and 2 show the results for scenarios with five studies where the probability of observing one outcome depends on both the observed covariates and the other outcome. The FCS approach that does not include the other endpoint (Fully Conditional 1) led to under‐coverage (below 0.9, Figure 1). This was anticipated because this imputation model is misspecified; it excludes the other outcome, which is predictive of missingness. The joint model provides joint CI coverage somewhat closer to the nominal levels than the correctly specified FCS approach (Fully Conditional 2), particularly for a higher proportion of missing data and strongly correlated outcomes within each participant. When we increased the strength of the study‐level correlation across outcomes (*ϕ* = 0.3), the CI coverage of Fully Conditional 2 deteriorated further, whereas that of the joint model remained close to nominal levels (Supporting Information, Figure 1). In terms of bias, both joint model and FCS approaches performed well on the continuous outcome treatment effect, with relative biases below 2% (Figure 2). However, the joint model produced the lowest bias in the binary outcome treatment effect. This approach also produced lower rMSE than the other methods (Supporting Information, Figure 2). We observed similar results when the continuous outcome is sporadically missing and the binary outcome is systematically missing (Supporting Information, Table 2).

## 6 Application to cardiac resynchronisation therapy data

The parameters of interest are the subgroup treatment effects (men vs women) on four outcomes: mortality and NYHA class (binary), 6‐min walk and quality‐of‐life (continuous). We fit models with a treatment‐by‐sex interaction and study‐level random intercepts (6). The latter accounts for differences in overall health of the populations across studies but assumes constant subgroup treatment effects. We have also considered models with study‐specific random treatment effects $\left(\beta_{1,j}^{k}∼N(0,\sigma_{\beta_{1}})\right)$ but focused our results presentation on the simpler models noting that models with more complex covariance structures led to similar results. The small number of studies and large number of outcomes made the parameters of the random effects variance–covariance matrix difficult to identify. To help improve convergence, we fixed the level‐2 covariances (off‐diagonal parameters) to zero across the different models. Level‐2 variance estimates were still imprecise, but convergence for these variances and level‐1 variance/covariance parameters was good (Gelman–Rubin factors smaller than 1.1).

We assumed that the outcomes followed a multivariate normal distribution, using latent normal variables for the two binary outcomes as described in Model (6). We assessed the statistical significance of the sex‐specific treatment effects by estimating simultaneous confidence/credible intervals and noting whether they excluded the null value. In the joint hierarchical model, the joint credible intervals were formed from the empirical posterior distribution; in the FCS–MI and CCA approaches, we used simultaneous confidence regions. Predictors of the missing data included all the variables in the substantive model (treatment, female and the interaction between them) plus other relevant predictors like baseline NYHA class and age.

(6) $$\begin{array}{cc} Z_{\mathrm{death},\mathrm{ij}} & ∼N(\mu_{1,\mathrm{ij}},1)\;\;\;\;P(\mathrm{deat}h_{\mathrm{ij}}=1)=P(Z_{\mathrm{death},\mathrm{ij}}>0)\\ Z_{\mathrm{nyha},\mathrm{ij}} & ∼N(\mu_{2,\mathrm{ij}},1)\;\;\;\;P(\mathrm{nyh}a_{\mathrm{ij}}=1)=P(Z_{\mathrm{nyha},\mathrm{ij}}>0)\\ \mathrm{wal}k_{\mathrm{ij}} & ∼N\left(\mu_{3,\mathrm{ij}},\sigma_{\mathrm{walk}}^{2}\right)\\ \mathrm{qo}l_{\mathrm{ij}} & ∼N\left(\mu_{4,\mathrm{ij}},\sigma_{\mathrm{qol}}^{2}\right) \end{array}$$

$$\mu_{k,\mathrm{ij}}=\beta_{k,0}+\beta_{k,1}\mathrm{CRT}\text{‐}D_{\mathrm{ij}}+\beta_{k,2}\mathrm{femal}e_{\mathrm{ij}}+\beta_{k,3}\left(\mathrm{CRT}\text{‐}D_{\mathrm{ij}}\times \mathrm{femal}e_{\mathrm{ij}}\right)+u_{k,\mathrm{ij}}+e_{k,\mathrm{ij}}$$

$$e_{k,\mathrm{ij}}∼N(0,\Omega_{e})u_{k,j}∼N(0,\Omega_{u})$$

**Figure 3:**
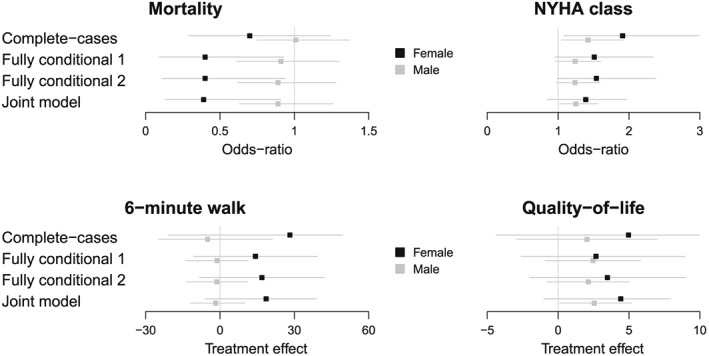
Subgroup treatment effects by gender on each outcome according to complete case analysis, fully conditional specification and joint model for addressing the missing data. All subgroup treatment effects favour CRT‐D, except men on 6‐min walk.

Figure 3 displays sex‐specific treatment effects for each outcome, according to method for addressing the missing data. Overall, compared with ICD alone, CRT‐D improved survival, quality‐of‐life and NYHA class. Women benefited substantially more from treatment on the 6‐min walk distance than men and also showed more pronounced survival benefits, although this difference (*β*_*k*,3_) was not statistically significant on any outcome, based on simultaneous intervals with 95% coverage. However, the individual intervals for the treatment effect interaction estimate did indicate statistically significant differences between men and women for mortality and 6‐min walk (Supporting Information, Figure 3). Men and women showed similar responses to treatment on quality‐of‐life and the NYHA improvement.

Complete‐case analysis produced a smaller difference between men and women on survival and a larger difference on 6‐min walk compared with the other methods. The joint model and fully conditional approaches led to similar point estimates of the differential treatment effect by sex, but the former reported somewhat smaller intervals. We note that the probability of each outcome being observed was weakly associated with the values of the other outcomes, so this application resembles the simulation scenarios where the joint model and FCS performed similarly. When we allowed a heterogeneous treatment effect across studies, the subgroup treatment effects and associated uncertainty remained fairly similar. With only five trials, the between‐study variance components were difficult to differentiate from zero.

## 7 Discussion

This paper compares two approaches for handling missing outcomes when combining individual patient data across studies. We found that the fully conditional approach compared well with the joint hierarchical model across most simulated scenarios and in the case study. The joint model had a small advantage in the mean squared error and CI coverage, particularly when the outcomes were strongly correlated and a large proportion of data was missing.

In a recent paper, He and Belin 19 compared a joint model and a fully conditional approach for dealing with (non‐hierachical) missing binary and continuous outcomes. The study shows that the two approaches perform similarly (irrespective of the order in which the variables were imputed under the FCS approach), although the joint model provided slightly better coverage in settings with smaller samples (*N* = 100). Our study extended these comparisons with a hierarchical setting (IPD meta‐analysis). Here, we might worry that the sequence of fully conditional models may not fully accommodate the correlation structure across outcomes. We found that when outcomes are strongly correlated and the probability of one outcome depends on the other outcome, the joint hierarchical model provides CI coverage consistently closer to nominal levels when compared with FCS–MI for estimating treatment effects. In addition, we consider a missing data pattern unique to hierarchical settings – outcomes may be completely missing for level‐2 units (in our example, the study). With systematically missing outcomes, we also found that the joint model slightly outperformed the fully conditional approach, even with small between‐study heterogeneity (e.g. intra‐cluster correlation of 0.1 and constant treatment effects).

In this paper we use a standard implementation of MI using chained equations previously considered for addressing missing predictors in IPD meta‐analysis 1. We believe that the current limitations of the FCS‐MI approach are mostly practical, and some improvements may be possible. For example, Carpenter and Kenward 14, p. 221‐2], suggest that, under an exchangeable correlation matrix (as assumed in our study), the univariate imputation model for each continuous outcome should include both mean and individual observations of the other outcomes (in this paper we have included only the latter). However, those results may not generalise to discrete outcomes. In addition, with a non‐exchangeable correlation matrix (e.g. different correlations between outcomes across studies), the conditional parameters may give information about the marginal covariance, threatening the validity of the fully conditional sampler 18.

Another potential extension would be to consider a probit model (instead of logistic model) to impute the binary outcomes. While in theory this might improve the convergence of the FCS algorithm to the posterior distribution implied by the joint hierarchical model, it is unclear the extent to which this would improve the performance of the FCS approach. For example, Carpenter and Kenward 14, p. 95] highlighted that although strictly uncongenial, in practice, we can impute a binary variable using a logit model and fit a probit model in the analysis, and vice versa.

While this study considered full‐Bayesian analysis for estimating the joint hierarchical model, this could be used with MI. An important feature of MI is that it allows the imputation model to be estimated separately from the analysis model, providing further flexibility when compared with full‐Bayesian analysis. First, there may be settings where we wish to include variables that are predictive of the missing values and the outcomes but are beyond those included in the model for the complete data. For example, post‐randomisation variables often provide important information about the missingness but should not be included in the analysis model 29. Second, combining data from very few studies, say less than five, causes computational problems for the joint hierarchical modelling. In these circumstances, a general recommendation is to use MI in a two‐stage IPD meta‐analysis 2. This would involve fitting a separate model to each study, and then combining the estimated parameters of interest using inverse‐variance weighted meta‐analysis. Under this approach, the imputation model would have to be implemented within study, and Rubin's rules would have to be applied to the multiple imputed estimates in each study, before undertaking the meta‐analysis 2. Third, while previous studies 13, 16, 30 found that approaches based on multivariate normality performed relatively well when missing outcomes are not normally distributed, there may be some cases where drawing from a posterior joint normal distribution may not be plausible, for example, if continuous outcomes are extremely skewed or semi‐continuous, such as hospital length of stay. With MI, before imputing the data, we can use a suitable (e.g. box‐cox) transformation to help make the normality assumption more plausible. The transformed outcomes are then multiply imputed and back‐transformed onto the original scale before applying the analysis model 15. With semi‐continuous outcomes, a two‐step imputation procedure may be preferred 31.

This study has some limitations. This paper considers missing outcomes only as the focus is on the relative merits of joint modelling and FCS when dealing with multiple incomplete outcomes. Obviously, one could have both outcomes and predictors missing for some of the studies. With several incomplete outcomes and predictors, full‐Bayesian modelling becomes increasingly challenging 16. For this reason, previous work addressing missing predictors in IPD meta‐analysis have advocated MI approaches 1, 2.

When designing a simulation study, the data generating process should avoid favouring any of the methods under comparison. Our simulation design may favour the joint hierarchical model because: (a) the outcomes are drawn from a joint distribution (using copulas) and (b) the covariance structure are explicitly partitioned into individual and study‐level components. We have considered an alternative data generating process (Supporting Information, Appendix D), which is more in line with the FCS approach. With the new simulation design, the performance of the FCS approach slightly improves (better CI coverage and reduced rMSE). However, the joint hierarchical model still provides consistently the lowest rMSE and coverage closest to nominal levels across the scenarios considered (Supporting Information, Tables 3 and 4).

The methods considered in this paper are not readily applicable to IPD meta‐analysis of longitudinal data. For example, imputing survival outcomes are not currently supported by multilevel MI using chained equations, although in principle this could be achieved by imputing under a Cox hierarchical regression model. While survival times could be jointly modelled with incomplete missing longitudinal outcomes 32, this would be complicated by data being clustered within studies.

Throughout the paper, we have assumed that the probability of observing one outcome was independent of its underlying unobserved values, conditional on the observed data (MAR). In practice, the true missingness mechanism is unknown given the data at hand. An assessment of the relative merits of the alternative approaches in addressing potential missing not at random mechanisms is underway. In this context, the use of selection models within a full‐Bayesian framework is appealing, in particular, when external information on the reasons for the missing data is available (e.g. expert opinion) to inform the sensitivity parameters via the priors 33. In general, we believe that accurately reflecting the correlation structure strengthens methods for handling incomplete correlated mixed outcomes in IPD meta‐analysis.

## Supporting information



Supporting Info Item
