# Supplementary material for: Handling incomplete correlated continuous and binary outcomes in meta‐analysis of individual participant data
Source: Stat Med. 2016 Apr 18;35(21):3676–89. doi: 10.1002/sim.6969 (PMC4982066; doi:10.1002/sim.6969)
Supplement: Supplementary file 1 — Supporting Info Item [file SIM-35-3676-s001.zip › WebAppendix_revised.pdf]

Supplementary material to

Handling incomplete correlated continuous and binary outcomes in meta-analysis of individual participant data

by Manuel Gomes, Laura Hatfield and Sharon-Lise Normand

Web Appendix A: MCMC Steps - Latent Normal Approach

We use superscripts to denote iterations, $r = 1, 2, ...,$ of the MCMC algorithm. To initiate the MCMC sampler, we replace the missing values by a random sample from the observed values, and choose initial values for $\boldsymbol{\beta}$, $\boldsymbol{\Omega}_e$ and $\boldsymbol{\Omega}_u$. Then at each iteration

Step 1. For $i = 1, ..., n$

a) Draw $Z^r_{ij} \sim N(\mu^{r-1}_{2,ij}, 1)$. If $Z^r_{ij} > 0$ set $Y^r_{2,ij} = 1$, otherwise set $Y^r_{2,ij} = 0$.

b) Draw $Y^r_{1,ij}$ from the conditional Normal given Z^r_{ij},

$$\text{N}\left\{\mu^{r-1}_{1,ij} + \left(Z^r_{ij} - \mu^{r-1}_{2,ij}\right)\rho^{r-1}\sigma^{r-1}_1, \left(1 - (\rho^{r-1})^2\right)(\sigma^{r-1}_1)^2\right\}$$

where $\mu_{k,ij} = \beta_{k,0} + \beta_{k,1}t_{ij} + \beta_{k,2}X_{ij} + u_{k,j}, \qquad k = 1, 2$

Step 2. Draw fixed coefficients $\boldsymbol{\beta}^r$ from the multivariate Normal distribution,

$$\text{MVN}\left[\left[\sum_{ij}\boldsymbol{\Lambda}^T\boldsymbol{\Omega}_e^{-1}\boldsymbol{\Lambda}\right]^{-1}\sum_{ij}\boldsymbol{\Lambda}^T\boldsymbol{\Omega}_e^{-1}(\boldsymbol{Y}_{ij} - \boldsymbol{u}_j)^T, \left[\sum_{ij}\boldsymbol{\Lambda}^T\boldsymbol{\Omega}_e^{-1}\boldsymbol{\Lambda}\right]^{-1}\right]$$

where $\boldsymbol{\Lambda} = \boldsymbol{I}_{2\times 2} \otimes \boldsymbol{X}_{ij}$, $\boldsymbol{Y}_{ij} = (Y_{1,ij}, Z_{ij})$, $\boldsymbol{X}_{ij} = (X_{1,ij}, X_{2,ij})$ and $\boldsymbol{u}_j = (u_{1,ij}, u_{2,ij})$

Step 3. Draw random coefficients $\boldsymbol{u}^r_j$ from

$$\text{MVN}\left[\left[\sum_{i}\boldsymbol{\Omega}_e^{-1} + \boldsymbol{\Omega}_u^{-1}\right]^{-1}\sum_{i}\boldsymbol{\Omega}_e^{-1}(\boldsymbol{Y}_{ij} - \boldsymbol{\beta}\boldsymbol{X}_{ij})^T, \left[\sum_{i}\boldsymbol{\Omega}_e^{-1} + \boldsymbol{\Omega}_u^{-1}\right]^{-1}\right]$$

Level-1 residuals can be easily obtained by subtraction, $\boldsymbol{e}^r_{ij} = \boldsymbol{Y}_{ij} - \boldsymbol{\Lambda}\boldsymbol{\beta} - \boldsymbol{u}_j$

Step 4. Update the elements of $\boldsymbol{\Omega}_e^{r-1}$ and $\boldsymbol{\Omega}_u^{r-1}$, in that order, and conditional on $\boldsymbol{Y}^r, \boldsymbol{\beta}^r, \boldsymbol{u}^r$, to obtain $\boldsymbol{\Omega}^r_e$ and $\boldsymbol{\Omega}^r_u$ [1].

Web Appendix B: Iterative Algorithm for Multiple Imputation using fully conditional specification

In this section we describe the steps of the Gibbs sampler for imputing missing data using fully conditional specification. The MI approach entails three main stages. First, the imputation model is fitted to the observed data. Second, we generate random draws of the unknown parameters given the values predicted in the first stage. Third, we replace (impute) the missing values of the outcomes using the values drawn in stage 2 and the observed data. More specifically and similarly to model (3) in the manuscript, let the imputation models for the continuous and binary outcomes be described as

$$\begin{aligned} Y_{1,ij}|Y_{2,ij}, \mathbf{W}_{1,ij}, u_{1,j} &\sim \mathrm{N}(\boldsymbol{\gamma}_1 \mathbf{W}_{1,ij} + \alpha_1 Y_{2,ij} + u_{1,j},\ \sigma_1^2) \\ Y_{2,ij}|Y_{1,ij}, \mathbf{W}_{2,ij}, u_{2,j} &\sim \mathrm{Ber}\left\{ \frac{exp(\boldsymbol{\gamma}_2 \mathbf{W}_{2,ij} + \alpha_2 Y_{1,ij} + u_{2,j})}{1 + exp(\boldsymbol{\gamma}_2 \mathbf{W}_{2,ij} + \alpha_2 Y_{1,ij} + u_{2,j})} \right\} \end{aligned} \tag{1}$$

where $\mathbf{W}_{k,ij}$ is the set of covariates predicting missingness and $u_k \sim N(0, \tau_k)$, for $k = 1, 2$. The unknown parameters (θ) for the imputation of the continuous and binary outcomes are $\boldsymbol{\theta}_1 = \{\gamma_1, \alpha_1, \tau_1, \sigma_1^2\}$ and $\boldsymbol{\theta}_2 = \{\gamma_2, \alpha_2, \tau_2\}$, respectively.

The aim is to impute the missing values in each outcome $(Y_{1,ij}^{mis}, Y_{2,ij}^{mis})$ from the posterior distribution under model (1) which can be written as

$$P(Y_{k,ij}^{mis}|\mathbf{W}_{k,ij}, Y_{-k,ij}, Y_{k,ij}^{obs}) = \int_{\theta_k} P(Y_{k,ij}^{mis}|\mathbf{W}_{k,ij}, Y_{-k,ij}, Y_{k,ij}^{obs}, \boldsymbol{\theta}_k) P(\boldsymbol{\theta}_k|\mathbf{W}_{k,ij}, Y_{-k,ij}, Y_{k,ij}^{obs}) d\boldsymbol{\theta}_k$$

where $Y_{-k,ij}$ indicates the outcome other than k, with $k = 1, 2$.
Given the initial values and standard priors [2], the rth iteration of the imputation procedure involves drawing successively from

Step 1.

a) Sample $\boldsymbol{\theta}_1^{(r)}$ from $p(\boldsymbol{\theta}_1|Y_{1,ij}^{obs}, Y_{2,ij}^{(r-1)}, \mathbf{W}_{1,ij})$

c) Sample $Y_{1,ij}^{mis^{(r)}}$ from $p(Y_{1,ij}^{mis}|Y_{1,ij}^{obs}, Y_{2,ij}^{(r-1)}, \mathbf{W}_{1,ij}, \theta_1^{(r)})$

Step 2.

a) Sample $\boldsymbol{\theta}_1^{(r)}$ from $p(\boldsymbol{\theta}_2|Y_{2,ij}^{obs}, Y_{1,ij}^{(r-1)}, \mathbf{W}_{2,ij})$

c) Sample $Y_{2,ij}^{mis^{(r)}}$ from $p(Y_{2,ij}^{mis}|Y_{2,ij}^{obs}, Y_{1,ij}^{(r-1)}, \mathbf{W}_{2,ij}, \theta_2^{(r)})$

Step 3. Typically after 10 to 20 iterations (sufficient to reach convergence), $Y_{1,ij}^{mis}$ and $Y_{2,ij}^{mis}$ are then imputed from the posterior distribution $p(Y_{1,ij}^{mis}|Y_{2,ij}^{*}, \mathbf{W}_{1,ij}, \boldsymbol{\theta}_1^{*})$ and $p(Y_{2,ij}^{mis}|Y_{1,ij}^{*}, \mathbf{W}_{2,ij}, \boldsymbol{\theta}_2^{*})$, respectively. $Y_{1,ij}^{*}$, $Y_{2,ij}^{*}$, $\boldsymbol{\theta}_1^{*}$ and $\boldsymbol{\theta}_2^{*}$ are the values drawn from the last iteration of the Gibbs sampler.

Web Appendix C: Code for implementing the joint Bayesian model (1) for two outcomes, one continuous and one binary.

```
model{

for (i in 1:N){     # N - No. of individuals

y1[i] ~ dnorm(mu1[i], tau.y1)     # Continuous response

mu1[i] <- beta[1,1] + u[cluster[i],1] +
beta[2,1]*treat[i] + beta[3,1]*x1[i] + beta[4,1]*x2[i]

y2[i] ~ dinterval(z[i],0) # Discrete response

z[i] ~ dnorm(mu2[i],sigma.y2)

mu2[i] <- beta[1,2] + u[cluster[i],2] +
beta[2,2]*treat[i] + beta[3,2]*x3[i] + beta[4,2]*x4[i]
+ alpha*(y1[i]-mu1[i])
}

# Study-level random effects

for (j in 1:J){ # J - No. of studies

u[j, 1:2] ~ dmnorm(zero, tau.u[1:2,1:2])

}

### PRIORS ###

# Level-2 covariance matrix
sigma.u1 ~ dnorm(0, 0.001)T(0,)
sigma.u2 ~ dnorm(0, 0.001)T(0,)
rho.u    ~ dunif(-1, 1)

# Level-1 covariance matrix
sigma.y1 ~ dnorm(0, 0.001)T(0,)
sigma.y2 <-1         #var(y2) is fixed to 1
rho      ~ dunif(0, 1)

# Betas
beta[1:4,1] ~ dmnorm(b0, B0[1:4,1:4])
beta[1:4,2] ~ dmnorm(b0, B0[1:4,1:4])

### NODE TRANSFORMATIONS ###

# Level-2 nodes
sigma2.u1<-sigma.u1*sigma.u1
```

```
sigma2.u2<-sigma.u2*sigma.u2
cov      <-sigma.u1*sigma.u2*rho.u

sigma2.u[1,1]<-sigma2.u1
sigma2.u[2,2]<-sigma2.u2
sigma2.u[2,1]<-cov
sigma2.u[1,2]<-cov

tau.u[1:2,1:2]<-inverse(sigma2.u[,])

# Level-1 nodes
sigma2.y1<-sigma.y1*sigma.y1
tau.y1   <-1/sigma2.y1
alpha    <-rho*(sigma.y1/sigma.y2)

}
```

Web Appendix D: Alternative data generating process.

Here we describe an alternative simulation design which generates the outcomes in two stages: 1) simulates study-level means, and 2) generates a distribution of individual-level outcomes for each study centred at those study-level means. At each stage, the continuous outcome is simulated first, and then the binary outcome is generated conditional on the continuous response as described in the model (2) below. By doing so, this approach is more in line with the fully-conditional specification.

Study-level means:

$$\begin{aligned} \phi_j^1 &\sim \mathrm{N}(\mu^1,\ \tau_1^2) \\ \phi_j^2 &\sim \mathrm{N}(\mu^2 + \theta(\phi_j^1 - \mu^1),\ \tau_2^2) \end{aligned} \tag{2}$$

Individual-level outcomes:

$$\begin{aligned} Y_{ij}^1, &\sim \mathrm{N}(\phi_j^1 + \beta_1^1 t_{ij} + \beta_2^1 X_{ij},\ \sigma_1^2) \\ Y_{ij}^2, &\sim \mathrm{N}(\phi_j^2 + \beta_1^2 t_{ij} + \beta_2^2 X_{ij} + \gamma(Y_{ij}^1 - \phi_j^1),\ \sigma_2^2) \end{aligned}$$

Study-level means (ϕ_j^1 and ϕ_j^2) are first simulated for each study j, and assumed to follow a Normal distribution centred at (μ^1, μ^2) and corresponding study-level variances (τ_1^1, τ_2^2). Model (2) allows the outcomes Y_{ij}^1 and Y_{ij}^2 to be correlated at the study level through the parameter θ, where $\theta = \psi(\tau_2/\tau_1)$. Outcomes for the ith individual are then simulated from Normal distributions centred at the previously generated study-level means (ϕ_j^1 and ϕ_j^2), and the corresponding individual-level variances (σ_1^2, σ_2^2). These outcomes are also allowed to be correlated at the individual level through the term γ, where $\gamma = \rho(\sigma_2/\sigma_1)$. Study-level and patient-level variances of the binary outcome (τ_2^2 and σ_2^2) are adjusted by $(1-\psi^2)$ and $(1-\rho^2)$, respectively. The proportion of the total variance at the study level is defined as $ICC = \tau^2/(\sigma^2 + \tau^2)$. After simulating Normal distributed Y_{ij}^1 and Y_{ij}^2, the latter is dichotomized into a binary outcome such that the $Pr(Y_{ij}^2 = 1) \simeq 0.2$ similarly to the data generating process described in section 4.1.

Results using this simulation design are reported in Web Tables 3 and 4.

References

[1] Browne WJ, "Mcmc algorithms for constrained variance matrices," *Computational Statistics and Data Analysis*, vol. 50, no. 7, pp. 1655–1677, 2006.

[2] Carpenter J and Kenward M, *Multiple Imputation and its Application*. Statistics in Practice, Chichester, UK.: Wiley, 2013.

Web Table 1

Table 1: Percent bias, rMSE, and CI coverage for the estimated treatment effect on continuous ($\beta_{1,1}$) and binary ($\beta_{2,1}$) outcomes across scenarios with five studies, and probability of observing one outcome depends on both the covariates and the other outcome (sporadically missing data). Study-level correlation is fixed across these scenarios $\phi = 0.1$.

			Bias (%)		rMSE		Joint CI
Correlation (rho)	% Missing	Method	$\beta_{1,1}$	$\beta_{2,1}$	$\beta_{1,1}$	$\beta_{2,1}$	Coverage
Low (0.2)	20	Full data	0.2	1.7	0.041	0.076	0.956
		Complete-cases	8.9	61.2	0.101	0.115	0.661
		Fully conditional 1	0.5	24.3	0.045	0.092	0.919
		Fully conditional 2	0.1	6.0	0.044	0.092	0.945
		Joint model	0.2	3.6	0.037	0.091	0.944
	50	Full data	0.2	1.7	0.041	0.076	0.956
		Complete-cases	10.2	150.6	0.137	0.171	0.650
		Fully conditional 1	1.1	121.8	0.054	0.114	0.904
		Fully conditional 2	0.2	43.9	0.053	0.109	0.931
		Joint model	0.1	8.4	0.039	0.082	0.954
High (0.7)	20	Full data	0.2	2.1	0.040	0.075	0.958
		Complete-cases	9.3	105.9	0.103	0.143	0.642
		Fully conditional 1	1.2	70.4	0.044	0.115	0.869
		Fully conditional 2	0.2	11.3	0.042	0.084	0.934
		Joint model	0.2	3.7	0.036	0.076	0.955
	50	Full data	0.2	2.1	0.040	0.075	0.958
		Complete-cases	11.4	153.8	0.141	0.206	0.635
		Fully conditional 1	1.9	121.0	0.054	0.159	0.812
		Fully conditional 2	1.2	24.7	0.050	0.102	0.913
		Joint model	0.7	6.1	0.036	0.081	0.954

Web Table 2

Table 2: Percent bias, rMSE, and CI coverage for the estimated treatment effect on continuous ($\beta_{1,1}$) and binary ($\beta_{2,1}$) outcomes across scenarios with five studies and Y1 is sporadically missing (MAR conditional on both covariates and the other outcome, and $\phi = 0.1$) and Y2 is systematically missing (MCAR).

Correlation (rho)	% Missing	Method	Bias (%)		rMSE		Joint CI
			$\beta_{1,1}$	$\beta_{2,1}$	$\beta_{1,1}$	$\beta_{2,1}$	Coverage
Low (0.2)	20	Full data	0.0	1.1	0.040	0.075	0.958
		Complete-cases	9.4	106.3	0.109	0.151	0.700
		Fully conditional 1	0.2	77.2	0.044	0.122	0.876
		Fully conditional 2	0.2	8.3	0.043	0.087	0.953
		Joint model	0.1	4.9	0.036	0.076	0.960
	50	Full data	0.0	1.1	0.040	0.075	0.958
		Complete-cases	12.5	150.6	0.158	0.243	0.647
		Fully conditional 1	0.3	122.3	0.053	0.186	0.824
		Fully conditional 2	0.1	44.1	0.050	0.141	0.912
		Joint model	0.1	8.4	0.037	0.083	0.957
High (0.7)	20	Full data	0.1	1.9	0.040	0.075	0.958
		Complete-cases	9.9	104.8	0.148	0.151	0.728
		Fully conditional 1	0.8	79.5	0.045	0.124	0.885
		Fully conditional 2	0.2	9.4	0.042	0.088	0.940
		Joint model	0.1	2.7	0.035	0.075	0.956
	50	Full data	0.1	1.9	0.040	0.075	0.958
		Complete-cases	13.1	137.6	0.233	0.962	0.683
		Fully conditional 1	0.9	117.2	0.045	0.233	0.869
		Fully conditional 2	0.6	40.6	0.043	0.097	0.915
		Joint model	0.2	5.0	0.036	0.080	0.960

Web Table 3

Table 3: Percent bias, rMSE, and CI coverage for the estimated treatment effect across scenarios reported in Web Table 1, but using an alternative simulation design (Web appendix D).

			Bias (%)		rMSE		Joint CI
Correlation (rho)	% Missing	Method	$\beta_{1,1}$	$\beta_{2,1}$	$\beta_{1,1}$	$\beta_{2,1}$	Coverage
Low (0.2)	20	Full data	0.1	1.2	0.040	0.075	0.953
		Complete-cases	8.4	46.3	0.100	0.109	0.714
		Fully conditional 1	0.9	16.0	0.046	0.092	0.921
		Fully conditional 2	0.5	6.9	0.045	0.089	0.940
		Joint model	0.4	3.1	0.040	0.085	0.947
	50	Full data	0.1	1.2	0.040	0.075	0.953
		Complete-cases	9.5	65.4	0.122	0.157	0.667
		Fully conditional 1	1.7	30.3	0.054	0.106	0.894
		Fully conditional 2	0.5	8.0	0.051	0.094	0.935
		Joint model	0.5	4.9	0.040	0.082	0.950
High (0.7)	20	Full data	0.2	1.9	0.042	0.076	0.954
		Complete-cases	10.3	78.7	0.101	0.113	0.659
		Fully conditional 1	2.1	42.8	0.050	0.097	0.873
		Fully conditional 2	0.8	10.6	0.045	0.084	0.931
		Joint model	0.4	3.9	0.041	0.077	0.948
	50	Full data	0.2	1.9	0.042	0.076	0.954
		Complete-cases	12.9	99.1	0.138	0.198	0.625
		Fully conditional 1	2.8	66.2	0.055	0.124	0.840
		Fully conditional 2	1.0	12.7	0.050	0.097	0.921
		Joint model	0.6	5.5	0.041	0.084	0.943

Web Table 4

Table 4: Percent bias, rMSE, and CI coverage for the estimated treatment effect across scenarios reported in Web Table 2, but using an alternative simulation design (Web appendix D).

			Bias (%)		rMSE		Joint CI
Correlation (rho)	% Missing	Method	$\beta_{1,1}$	$\beta_{2,1}$	$\beta_{1,1}$	$\beta_{2,1}$	Coverage
Low (0.2)	20	Full data	0.1	1.4	0.040	0.075	0.953
		Complete-cases	9.0	76.0	0.101	0.132	0.755
		Fully conditional 1	1.7	52.1	0.047	0.118	0.882
		Fully conditional 2	0.4	6.8	0.045	0.093	0.948
		Joint model	0.3	5.7	0.040	0.081	0.956
	50	Full data	0.1	1.4	0.040	0.075	0.953
		Complete-cases	12.1	90.2	0.119	0.197	0.698
		Fully conditional 1	1.1	65.8	0.056	0.145	0.854
		Fully conditional 2	0.6	23.3	0.051	0.119	0.936
		Joint model	0.4	7.0	0.043	0.087	0.943
High (0.7)	20	Full data	0.2	2.3	0.042	0.076	0.954
		Complete-cases	10.9	99.8	0.127	0.134	0.781
		Fully conditional 1	1.5	61.4	0.048	0.112	0.861
		Fully conditional 2	0.7	14.1	0.046	0.095	0.942
		Joint model	0.3	6.3	0.042	0.079	0.947
	50	Full data	0.2	2.1	0.042	0.076	0.954
		Complete-cases	16.4	111.5	0.202	0.393	0.667
		Fully conditional 1	2.1	90.9	0.047	0.104	0.824
		Fully conditional 2	0.9	15.7	0.045	0.097	0.919
		Joint model	0.5	7.6	0.041	0.082	0.939

Web Figure 1

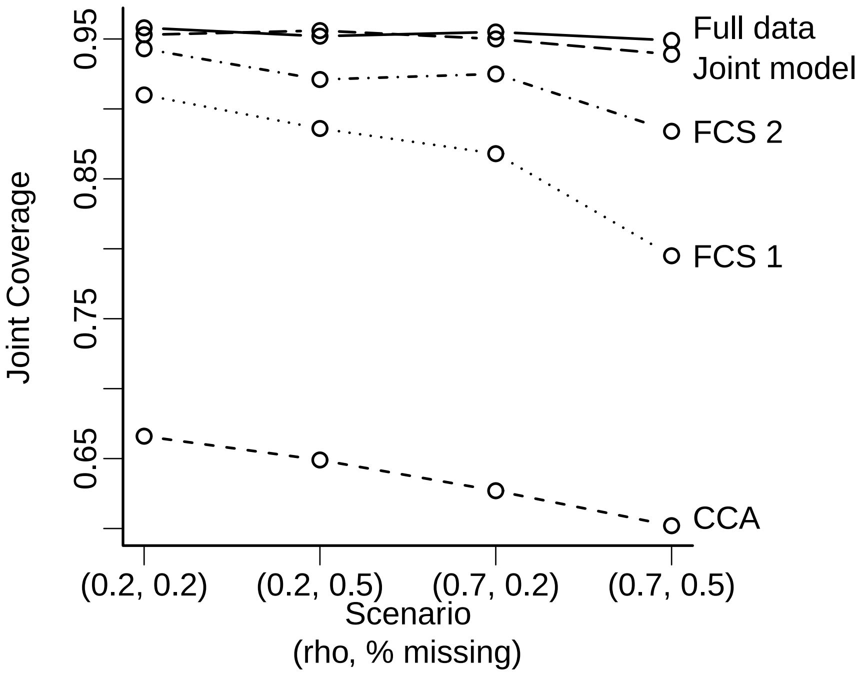

Figure 1: Joint CI coverage of treatment effects on both outcomes when the probability of observing one outcome depends on both the covariates and the other outcome (sporadically missing data), for higher levels of study-level correlation: $\phi = 0.3$. The lines are used to improve visualisation but do not reflect an increase of a single parameter in the X-axis. FCS: fully conditional specification; CCA: complete-case analysis.

Web Figure 2

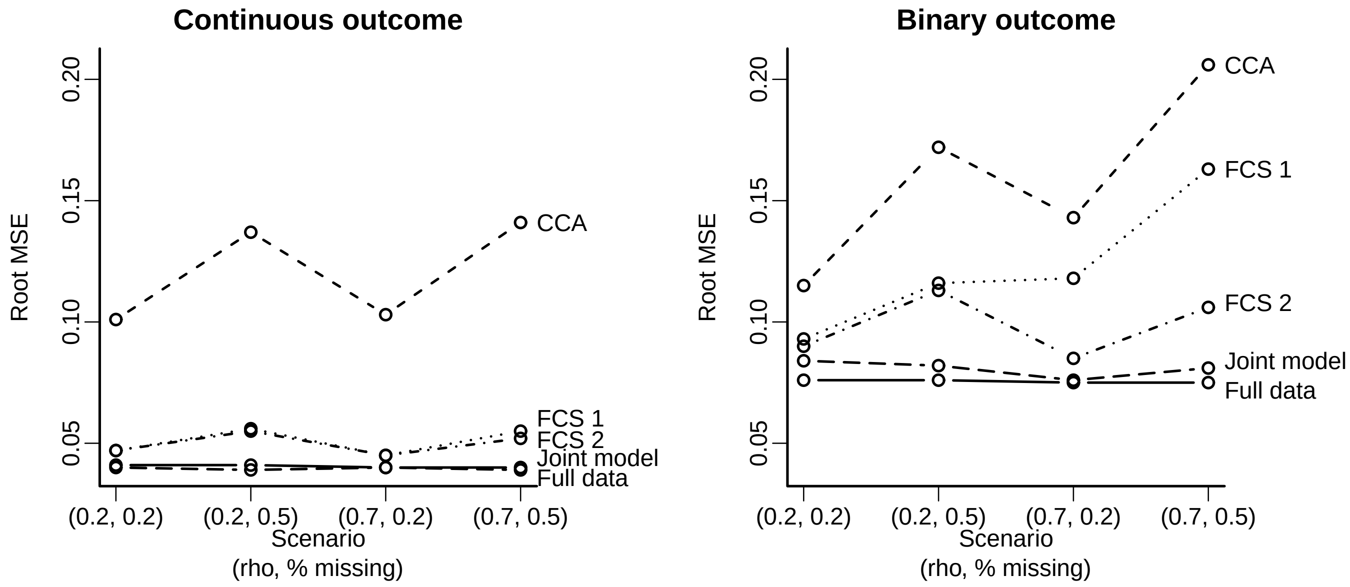

Figure 2: Root mean square error from estimating treatment effects on both continuous and binary outcomes according to method, for scenarios where the probability of observing one outcome depends on both the observed covariates and the other outcome (sporadically missing outcomes). Study-level correlation is fixed across these scenarios: $\phi = 0.1$. The lines are used to improve visualisation but do not reflect an increase of a single parameter in the X-axis. FCS: fully conditional specification; CCA: complete-case analysis.

Web Figure 3

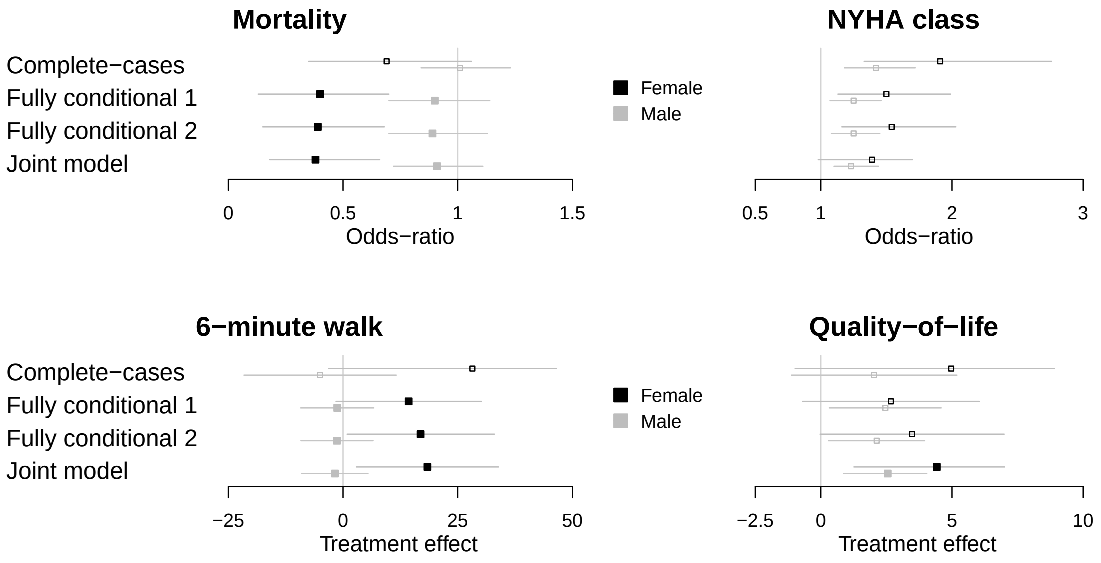

Figure 3: Subgroup treatment effects by gender on each outcome according to complete-cases, fully conditional specification and joint model for addressing the missing data. Filled (clear) marker indicates treatment by gender interaction is (not) statistically significant at 5% level, based on individual confidence intervals for each parameter.
